# Toward an Emerging Public Health Paradigm: Agriculture and Food Production for Health

**DOI:** 10.3390/foods15030527

**Published:** 2026-02-03

**Authors:** Rod Wallace, Katherine Frels, Maria Itria Ibba, Conrad Lyford, Devin Rose, David Baltensperger, Jan A. Delcour, Steven Greenspan, Alison Lovegrove, Barbara Schneeman, Peter Shewry, Edward Souza, William W. Wilson, Gary W. Yohe, Jim Anderson, George Annor, Jayne Bock, Claudia Carter, Brett Carver, Jianli Chen, Edward C. Deehan, Noah DeWitt, Lisa Diewald, Jason Donovan, Corrine K. Hanson, David Holding, Amir Ibrahim, Mariah Jackson, Sarah W. Kariuki, Elisa Karkle, Margaret Krause, Silvenus O. Konyole, Shuyu Liu, Jayson Lusk, Mohsen Mohammadi, Therese Narzikul, William Nganje, Gulnihal Ozbay, Ali Parsaeimehr, Andrew Ross, Jackie Rudd, Rachel Schendel, Rebecca Shenkman, Yong-Cheng Shi, Senay Simsek, Mark Sorrells, Payam Vahmani, Devin Wallace, Jochum Wiersma, Keona Wynne, Guorong Zhang, Xiaofei Zhang, P. Stephen Baenziger

**Affiliations:** 1 Foundation for Innovation in Healthy Food, 1407 N Bancroft Pkwy, Wilmington, DE 19806, USA; rod.wallace@fihf.org (R.W.);; 2 Department of Agronomy and Horticulture, University of Nebraska-Lincoln, 202 Keim Hall, Lincoln, NE 68583, USA; 3 Centro Internacional de Mejoramiento de Maíz y Trigo (CIMMYT), C.A.P. Plaza Galerías, Col. Verónica Anzures, Ciudad de Mexico 11305, Mexico; 4 Department of Agricultural and Applied Economics, Texas Tech University, 2500 Broadway, Lubbock, TX 79409, USA; 5 Department of Food Science and Technology, Nebraska Food for Health Center, University of Nebraska-Lincoln, Lincoln, NE 68588, USA; 6 Soil and Crop Sciences Department, Texas A&M University, 6370 Olsen Blvd. TAMU 2474, College Station, TX 77843, USA; 7 Department of Food Chemistry and Biochemistry, KU Leuven, Kasteelpark Arenberg 20, 3001 Leuven, Belgium; 8 Rothamsted Research, West Common, Harpenden AL5 2JQ, UK; 9 Nutrition Department, University of California Davis, 3135 Meyer Hall, One Shields Avenue, Davis, CA 95616, USA; 10 Department of Plant Pathology, Kansas State University, 1301 Mid Campus Drive, Manhattan, KS 66506, USA; 11 Department of Agribusiness and Applied Economics, North Dakota State University, NDSU Dept. 7610, Fargo, ND 58108, USA; 12 Department of Economics and Environment, Wesleyan University, 318 High St., Middletown, CT 06457, USA; 13 Department of Agronomy and Plant Genetics, University of Minnesota, 411 Borlaug Hall, 1991 Buford Circle, St. Paul, MN 55108, USA; 14 Department of Food Science and Nutrition, University of Minnesota, 1334 Eckles Avenue, St. Paul, MN 55108, USA; 15 Wheat Marketing Center, 1200 NW Naito Parkway, Suite 230, Portland, OR 97209, USA; 16 California Wheat Commission, 1240 Commerce Avenue, Suite A, Woodland, CA 95776, USA; 17 Department of Plant and Soil Sciences, Oklahoma State University, 209F Noble Research Center, Stillwater, OK 74078, USA; 18 Aberdeen Research & Extension Center, University of Idaho, 1693 S 2700 West Rd., Aberdeen, ID 83210, USA; 19 School of Plant, Environmental and Soil Sciences, Louisiana State University, Baton Rouge, LA 70803, USA; 20 MacDonald Center for Nutrition Education and Research, Villanova University, 800 Lancaster Avenue, Villanova, PA 19085, USA; 21 International Development Research Centre (IDRC), Juncal 1385, Piso 14, 11000 Montevideo, Uruguay; 22 Department of Medical Sciences, University of Nebraska Medical Center, 984045 Nebraska Medical Center, Omaha, NE 68198, USA; 23 Centro Internacional de Mejoramiento de Maíz y Trigo (CIMMYT), P.O. Box 1041 Village Market, Nairobi 00621, Kenya; 24 Department of Crop and Soil Science, Oregon State University, 3050 SW Campus Way, Corvallis, OR 97331, USA; 25 Department of Nutritional Sciences, Masinde Muliro University of Science and Technology, Kakamega P.O. Box 190-50100, Kenya; 26 Department of Agricultural Economics, Oklahoma State University, Stillwater, OK 74078, USA; 27 Department of Agronomy, Purdue University, 915 Mitch Daniels Blvd, West Lafayette, IN 47907, USA; 28 Department of Agriculture and Natural Resources, Delaware State University, 1200 North DuPont Highway, Dover, DE 19901, USA; 29 Department of Biology and Microbiology, South Dakota State University, Brookings, SD 57007, USA; 30 Department of Animal and Food Science, University of Kentucky, 410 W.P. Garrigus Building, Lexington, KY 40546, USA; 31 Department of Grain Science & Industry, Kansas State University, 211 Shellenberger Hall, Manhattan, KS 66506, USA; 32 Department of Food Science, Purdue University, 745 Agriculture Mall Dr., West Lafayette, IN 47907, USA; 33 Section for Plant Breeding and Genetics, School of Integrative Plant Science, Cornell University, 240 Emerson Hall, Ithaca, NY 14853, USA; 34 Department of Animal Science, University of California Davis, One Shields Ave, Davis, CA 95616, USA; 35 Electrical and Systems Engineering, Washington University in St Louis, One Brookings Drive, St Louis, MO 63130, USA; 36 Department of Agronomy and Plant Genetics, Northwest Research and Outreach Center, University of Minnesota, 2900 University Avenue, Crookston, MN 56716, USA; 37 Agricultural Research Center-Hays, Department of Agronomy, Kansas State University, 1232 240th Ave, Hays, KS 67601, USA; 38 Department of Plant Sciences, University of California Davis, One Shields Avenue MS4, Davis, CA 95616, USA

**Keywords:** economics, dietary fiber, nutrition policy, health-promoting agriculture, chronic disease prevention, fiber enriched wheat, food systems

## Abstract

An emerging paradigm in public health focuses on enhancing nutrition in existing food staples to reduce chronic disease at the population scale, rather than relying on individuals to change their behavior. This paradigm leverages plant and animal breeding, production practices, and processing to enhance nutrition, whereby foods consumed by millions can be improved at low incremental cost. This article supports and operationalizes this paradigm, illustrating the potential to improve diets through a case study that increases the arabinoxylan fiber content of commodity wheat through classical plant breeding (a non-GMO technology). The approach described in this article proposes to link agricultural and food science with health system implementation to deliver equitable access, improved healthcare outcomes and cost savings, and improved community health. Based on published dose–response relationships, comparative risk modeling indicates that modest fiber increases achieved by the commodity wheat breeding led to reduced population-level risks of 1–3% for cardiovascular disease, 3–4.5% for type 2 diabetes, and 1–3.5% for colorectal cancer, translating into substantial healthcare cost savings when implemented at a national scale. This article outlines possible low-risk pathways for implementing these nutrition increases at the population scale through commodity supply chains and community-level nutrition improvement efforts and evaluates the ranges of potential population-level impacts.

## 1. Introduction

Chronic disease, driven partly by poor diet choices, burdens the global economy and strains healthcare systems. Increasing fiber intake could be an important part of addressing the problems. The Global Burden of Disease 2023 study, assessing dietary habits in 195 countries, found that in all regions evaluated, dietary fiber intake was below that study’s target of 25 g per day [1]. Deficiencies across high-income countries globally [2] are illustrated by the U.S., where only 5% of the population consumes that nation’s recommended intake of dietary fiber intake per day (25 g for women and 38 g for men, age 19–50 years old; 21 and 30 g, respectively, over age 50) [3]. This underconsumption of dietary fiber is estimated to contribute to annual American healthcare and related costs of USD 4516–10,624 per person [4]. Obesity is the fifth most common cause of disease burden in the middle-income nations of Latin America, Eastern Europe, and Asia. One reflection of this trend is the increasing consumption of lower-fiber grains, in the processed form of bread and pasta, in place of traditional, higher-fiber grains such as millet and sorghum, as well as root crops [5]. Lower-income countries, meanwhile, have also been transitioning to lower-fiber diets. In sub-Saharan Africa, demographic changes including rapid urbanization, rising incomes, and more job opportunities for men and women have shifted diets towards more convenient and lower-fiber foods [6].

In pursuit of healthier outcomes, modern health systems provide quality dietary guidelines (e.g., [7,8]) that individuals could use to increase their fiber intake through several approaches. For example, individuals could shift their diet from foods without fiber, such as animal-based foods and sugars, towards more fruits and vegetables. People could replace refined grains with whole-grain options or incorporate more fiber-dense foods, such as legumes, into their diets. However, in this individual-focused framework, each person must be uniquely coached to follow such advice, which is difficult to cost-effectively scale and appears to be insufficient. Meanwhile, food production and processing are typically characterized by large-scale production and modest profit margins [9] and there are relatively few efforts to systematically target them to enhance nutritional composition and end-user benefits [10].

**Figure 1:**
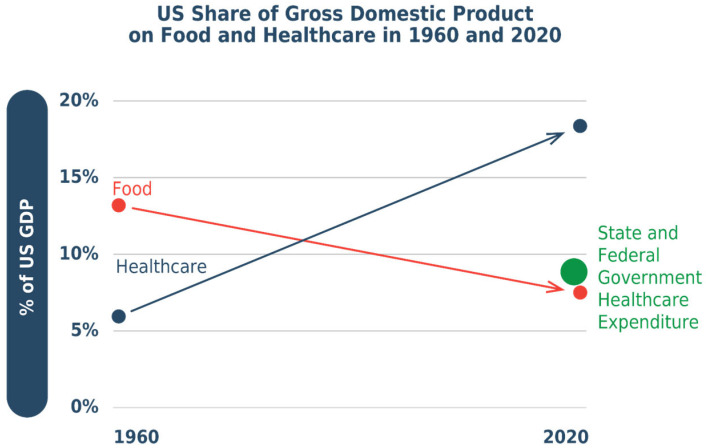
U.S. food and healthcare expenditures as a share of gross domestic product (GDP) in 1960 and 2020. Data sources: GDP from the U.S. Bureau of Economic Analysis (BEA) National Income and Product Accounts; healthcare expenditure and national share from the Centers for Medicare and Medicaid Services; food expenditure from the Bureau of Economic Analysis.

To provide a historical perspective, Figure 1 illustrates how the economic situation has evolved over time for food and healthcare, using the U.S. experience to illustrate.

Since 1960, the value of healthcare has grown as a share of the economy, while the value of food and agriculture has shrunk. Today, America spends twice as much on healthcare as on food and agriculture—that ratio having flipped over the past sixty years [11]. The U.S. and state government spending on healthcare (USD 6500 per person, 48% of total healthcare spending) surpasses consumer spending on food (USD 6400 per person annually).

This article aims to articulate and evaluate an emerging public health paradigm that could shift the trend. The paradigm can be identified as the HEAL approach, for Health Embedded Agriculture and Logistics. It integrates agricultural innovation, food processing, and health system incentives to improve population nutrition at scale, using increased arabinoxylan fiber in commodity wheat as a case study.

This approach was developed using an integrated, interdisciplinary analysis of the existing agriculture, food, and health systems that shape nutritional outcomes. The analysis draws on evidence from plant breeding and production practices to work toward improving the nutritional quality of commodity crops and animals, processing practices to enhance nutrition, and economic mechanisms to align incentives across food and healthcare systems to support public health objectives [12].

Section 2 describes the food technologies that enhance nutrition at the global scale in foods that consumers currently enjoy. Section 3 outlines two approaches that apply such nutrition enhancements by aligning financial incentives across current socioeconomic systems that deliver our food and manage our health. Section 4 discusses potential health impacts, and Section 5 illustrates how this emerging paradigm can be executed, relying on technical know-how and capable food and healthcare systems that already exist. To implement the ideas in the following sections, coordination and commitment are required, which are unfortunately currently missing.

## 2. Food Technologies That Enhance Nutrition and Population Health

**Figure 2:**
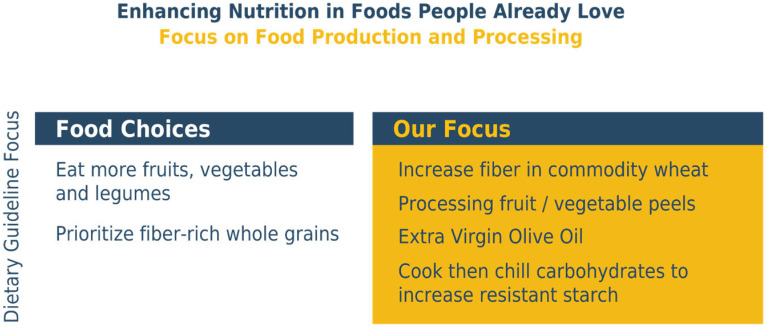
On the left are food choices that consumers can make to further align their diets with guidelines. The right side illustrates examples of food production and processing choices that can increase nutrients in many individuals’ current diets.

Figure 2 illustrates existing technologies that fit this article’s emerging paradigm.

The recent literature illustrates the diversity of such technologies, for example, the potential to reduce the intake of trans fatty acids, saturated fats, and sugars by reformulating existing foods [13,14], or increasing micronutrient intake by targeted plant breeding and growing techniques [15]. Other technologies can also improve nutrition, while retaining other aspects of perceived food and ingredient quality. For example, cooling foods based on rice [*Oryza sativa*], wheat [*Triticum* spp.], corn [*Zea mays*], or potato [*Solanum Tuberosum*] after cooking increases prebiotic resistant starch, which acts similarly to fiber [16]), even if then reheated.

Opportunities exist for modest changes in the nutrient composition of agricultural commodities that retain taste and other quality characteristics [17]. Changes in temperature and rainfall, for instance, change the biochemical composition of crops, and food producers must be able to handle this variation. This ability to manage environmentally induced change can also support the ability to manage changes that improve a commodity’s nutritional content, while maintaining foods’ organoleptic properties.

In a previous paper, several of this article’s authors outlined the scientific potential of increasing dietary fiber levels in wheat-based foods—the source of 20% of global calories and protein and 17% of American diets [18]. The core evidence was provided by the UK Biotechnology and Biological Sciences Research Council’s Delivering Sustainable Wheat program, which illustrates that commercial wheat cultivars vary in arabinoxylan fiber content. This cell wall polysaccharide is the main component of dietary fiber in both whole wheat grain and white flour. Arabinoxylan is the primary fiber component of endosperm cell wall tissue and can be increased by selective breeding. The arabinoxylan in flour is either water-extractable or water-unextractable. The water-unextractable form has deleterious impacts on the bread-making quality of wheat flour, probably because of its interference with the gluten network in the dough. Here, it is common industrial practice to use xylanase enzymes, which render the water-unextractable arabinoxylan extractable, thereby significantly increasing bread volume [17].

To illustrate how increased-fiber wheat can be commercially successful, it is important to know that cultivar sources of increased-fiber genes were successfully commercialized in the UK, France, and China before anyone knew that they were higher in fiber. These cultivars provide high farming yields, good bread-making quality, and improved nutrition [19]. A similar fiber variation is identified in high-quality American cultivars. Farmers choosing to increase the production share of comparatively increased-fiber wheat cultivars would increase the fiber in the value chain (without reducing yield), and hence the fiber intake across populations, without relying on consumers to change their food choices.

**Figure 3:**
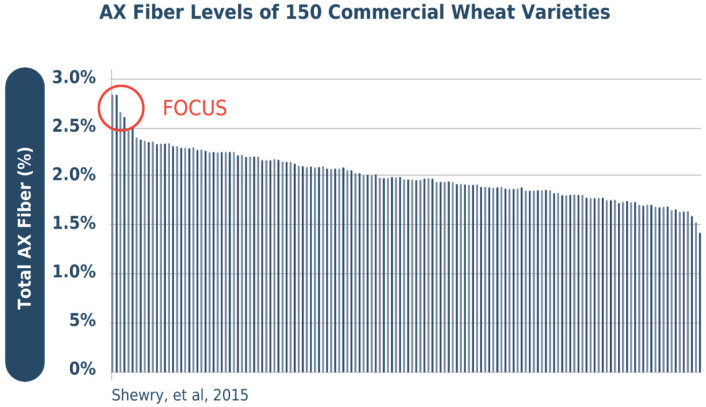
This figure [21] charts variation in arabinoxylan fiber levels among commercial wheat cultivars. Each bar across the horizontal access is a commercial variety, with Y axis as the total percent arabinoxylan fiber (AX). A few (under 10%) ancient varieties or other grains are included, without impacting takeaways.

In the future, the application of classical plant breeding technology could further increase wheat’s dietary fiber levels. Cultivars with higher dietary fiber can be—and are being—developed, based on research at Rothamsted Research [10], across market classes and geographies. Scientists are extending 19 years of research on the impact of arabinoxylan on wheat production, composition, and health impacts, to ensure that they will maintain bread-making quality, taste, and affordability [18]. Their ongoing studies show that modern elite wheat cultivars vary by up to 2-fold in arabinoxylan content in white flour (see Figure 3) and that further increases should be achieved by selective breeding. Past and ongoing efforts to reduce micronutrient deficiencies through wheat and other staple foods have been underway in the Global Alliance for Improved Nutrition HarvestPlus initiative [10,20] and other Consultative Group on International Agricultural Research (CGIAR) programs.

Conducted at scale, the strategy of improving the nutrient profile of commonly consumed foods is projected to deliver public health benefits across millions of people of all socioeconomic groups. With an average fiber intake increase of up to 2.5 g per day as a result of just increasing the arabinoxylan fiber content of commodity wheat, two standard deviations around mean forecasts reduced the risk by 1–3% for cardiovascular disease [22], 3–4.5% for type 2 diabetes [22,23], and 1–3.5% for colorectal cancer [24]. Moreover, an increased intake of dietary fiber is also recognized as improving health outcomes for individuals who are already afflicted by chronic conditions [25], with such benefits received virtually contemporaneously with the intervention.

**Figure 4:**
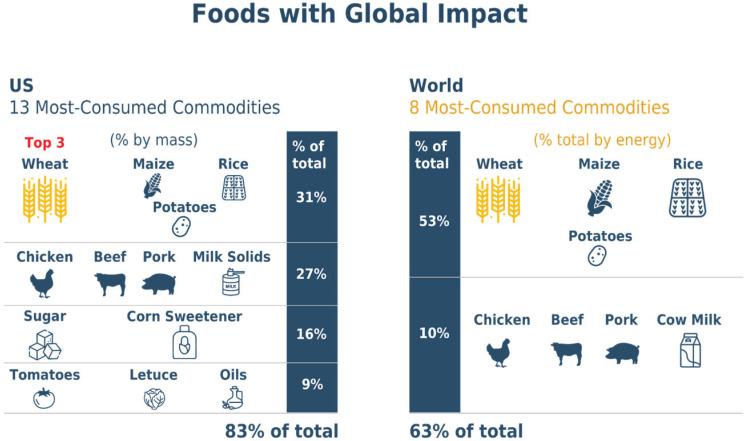
Illustrates the importance of a handful of commodities in our diets. On the left are the most consumed commodities as a percentage of the mass of U.S. food production for human consumption, based on U.S. Department of Agriculture Ag and Food Statistics. On the right are the most consumed commodities globally, organized by percent of energy intake, based on United Nations Food and Agriculture Organization data.

This paradigm can be extended to scientific opportunities to improve nutritional profiles beyond wheat. As illustrated in Figure 4, with just a handful of crops and animals providing nearly two-thirds (63%) of global food consumption (wheat, rice, corn, chicken [*Gallus gallus domesticus*], cattle [*Bos taurus*], swine [*Sos scrofa domesticus*], and dairy [mainly from *B. taurus*]), enhancing nutrients in these staple foods could improve health broadly. These commodities all naturally vary along many dimensions, and promising opportunities for enhancing nutrients exist in these staples [26,27].

## 3. Methods: Enhancing Foods and Aligning Incentives to Add Value

Meta-analysis and a comparative risk assessment framework are used to estimate the potential health and economic impacts of increased dietary fiber intake, incorporating baseline disease incidence, plausible adoption rates, and healthcare costs, with uncertainty explored using probabilistic modeling implemented in Lumivero’s @RISK 8.12 software (https://help-risk.lumivero.com/v8_12/en/Home.htm) [28].

Section 3 presents two complementary implementation pathways to operationalize the proposed science at the population scale. The first pathway leverages the food industry’s high-volume supply chains to deliver nutrition enhancements broadly across the population at the lowest cost. The second targets nutrition-based benefits at the community level.

These approaches treat food and healthcare as an integrated system [29,30], offering complementary routes to improve population nutrition. Both options can be implemented with a high probability of success as a result of low implementation cost, combined with an emphasis on mutually benefitting all stakeholders, with financial incentives applied at key influence points.

**Figure 5:**
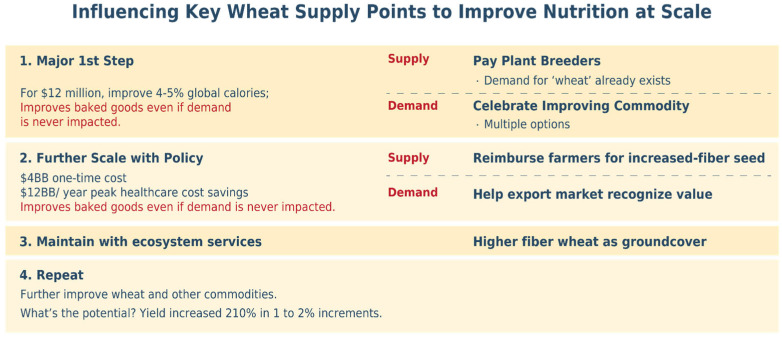
A four-stage implementation approach to improve commodity wheat quality.

Figure 5 summarizes a step-by-step implementation approach for increased-fiber wheat. The top section outlines stage 1, Major 1st Step: the left column provides background data illustrating the potential to pay a small number of wheat breeders to influence the food supply at scale, even if demand specifically for wheat fiber is never created. The right-hand column illustrates the primary, supply-driving activity and the consumer demand-focused activities that can accelerate the effort. Stages 2, 3, and 4 follow similar patterns.

### 3.1. Enhancing Crop Nutrients for Global Impact

The first approach inexpensively expands crop nutrient improvement programs into systematic processes. Some low- and middle-income nations currently have systems to financially reward farmers for increasing crop nutrition. Ghana’s “Planting for Food and Jobs” program, for example, incentivizes the growth of fortified crops and the adoption of better agricultural practices. High-income nations such as the U.S. also have implemented programs in which the government financially subsidizes socially beneficial farming practices, including by paying for seeds [31,32,33].

In Figure 5’s illustrative Stage 1 for increased-fiber wheat, an estimated USD 12 million effort over 5 years (An additional 2–3 years is required to multiply the seed volume of resulting varieties, with no regulatory approval required) kick-starts systematic global change, creates increased-fiber varieties, and begins increasing the nutritional quality of commodity wheat. A few hundred plant breeding professionals worldwide determine much of the future of wheat’s 20% of global food supply in labs that could easily implement—at modest incremental cost—the fiber targets discussed in this paper. That USD 12 million investment is enough for the breeders of the majority of wheat cultivars for low- and middle-income nations, United Nations Food and Agriculture Organization CIMMYT, to introgress targeted, increased-fiber wheat genes into their most productive cultivars across their target geographies. Over time, the nutritional quality of CIMMYT wheat cultivars, delivering approximately 4% of the world’s calories, would be improved.

Estimated budgets suggest that the initial USD 12 million investment would also fund the breeding of increased-fiber wheat cultivars together with the most productive U.S. wheat cultivars, covering half of American wheat-producing geographies. This initial breeding effort would accelerate progress towards traditional wheat breeding goals of feeding the world by reducing food costs and increasing production yields. Developing increased-fiber cultivars would also increase the average nutritional quality of U.S. wheat (6–7% of global wheat production, and 17% of American diets [34]), even if demand specifically for the increased-fiber wheat characteristic never materializes. This increased-fiber wheat can be sold in the current supply chain as “commodity wheat” (blended with other cultivars), satisfying current wheat demand based on their non-nutritional characteristics. The author team estimates that a decade would be required to achieve peak market penetration for these cultivars, based on common uptake patterns for new wheat varieties, unless further actions were taken to develop the market for increased-fiber wheat.

There are also opportunities to accelerate and expand planting of increased-fiber wheat during this Stage 1. The first such opportunity begins immediately. Commercial wheat varieties naturally vary in dietary fiber content, and those with relatively higher content can be targeted, to be replaced later by varieties bred to have even higher fiber. The neoclassical microeconomics curriculum illustrates that when actions can be taken that would deliver value to society—such as by improving the health of millions of individuals—that value will accrue to stakeholders across society. Market opportunities will exist in which firms can capture part of the value as profit by spurring stakeholders that value public health to financially support supply of these services. Many such marketing opportunities related to increased-nutrient foods remain under-explored. For instance, targets set by major retailers (up to 24% of grocery sales [35]) to increase the nutritional quality of existing baked goods, and communication by brands about the effort to deliver “new and improved” nutritional quality for buyers and their communities, can support demand growth that benefits large communities. These benefits and marketing opportunities harken to memories of fortified foods, such as flour enriched with B-vitamins, with marketing campaigns that celebrated their health benefits [36]. The incremental cost to supply such demand can be minimized by also blending increased-fiber wheat into the commodity food supply chain, aiming to benefit the entire population by enhancing average nutritional quality. Branded organizations may also choose to separate or “identity preserve” increased-fiber wheat, marketing it as a wholly new ingredient, which may, or may not, be carried out at a large scale. Consumer education on nutrition, in K-12 schools, may further enhance demand.

The focus of Stage 2 is accelerating the adoption of increased-fiber cultivars to the population scale, primarily supported by policy that financially rewards farmers growing increased-fiber cultivars, leading to broad national and international delivery of increased-fiber wheat. Financial rewards can be in the form of reimbursement for seed purchases by farmers who choose to grow certified, increased-fiber wheat cultivars. Analysis predicts that government savings in reduced healthcare expenses for chronic diseases and better healthcare outcomes would be considerably larger than the financial incentive paid to farmers.

In the case of increased-fiber wheat, a U.S. program described in the meta-analysis below would incur an initial cost of estimated USD 4.2 billion spread over 7 years, for projected ongoing healthcare cost savings of USD 12 billion annually (USD 84 billion over 7 years as described in detail below). With only a few thousand farmers growing 50% of U.S. wheat [34], the program could be scaled at modest cost. The value of this incentive program could be extended and market impact accelerated by programs that educate wheat buyers outside the U.S. about the nutritional value of increased-fiber wheat, further increasing sales and thus health benefits. With transparent financial incentives that are material for farmers, supporting implementation, the probability of success would be high. Similar programs can be executed in other countries.

In Stage 3, the paradigm focuses on maintaining increased-fiber wheat production. By this stage, demand would have driven wheat breeders to introgress increased-fiber genes into the bulk of the best cultivars, and increased-fiber traits would have become the norm in plant breeding populations. As feasible as it is to breed increased-fiber cultivars, this progress can be lost without ongoing efforts. Actions need to be taken to ensure that the genes supporting increased-fiber levels remain in breeding populations. While required, simulations suggest that even relatively modest, nonprofit-driven efforts would be sufficient to convince plant breeders to continue to select and retain increased-fiber genes in their breeding pool. For instance, many nonprofit organizations currently influence farmers’ seed selection, notably to enhance farmer incomes or reduce ecological impact. Wheat is an excellent cover crop [37], helping to improve soil quality and health while reducing erosion and chemical runoff. Healthier cultivars can be promoted for such cover crop use, highlighting benefits for farmers, ecosystems, and human health.

In Stage 4, the process repeats. Other increased-nutrient wheat characteristics and processing choices can follow.

The food industry has proven successful with such step-by-step, “stacked” innovation delivered at scale. Since 1950, U.S. wheat yield, for instance, has increased a seemingly modest 1 to 2% a year—yet the total across those years is an impressive 210% increase. Similar 75-year nutrition changes can add up, especially if each year’s change saves billions of annual healthcare dollars.

### 3.2. Community Food Supply Intervention

The second approach links enhanced nutrition to healthcare insurance at the community scale, rather than focusing on where healthcare professionals tend to focus: individual-by-individual healthcare. Programs focused on influencing consumer choice at this point in the supply chain are relatively common: see, for example, the Dutch Supreme Nudge and supermarket sales programs [38,39], and South Africa’s Healthy Food rebate [40]. Existing offerings at foodservice locations can also be cost-effectively influenced to improve community health at low risk.

#### 3.2.1. Delivering Community Food Supply Intervention

Delaware State University’s foodservice, feeding approximately 7000 students, faculty, and staff, illustrates the potential reach of community-focused interventions. Based on demographic- and socioeconomic-adjusted prevalence estimates, this is a sizable at-risk population, with roughly 3600–5000 cases of dysglycemia (prediabetes or type 2 diabetes) and elevated cardiovascular disease risk (Table 1) [41,42,43,44,45,46,47,48].

Table 1 shows the estimated prevalence ranges for selected cardiometabolic conditions and risk categories in the target population of 6000 students and 1000 faculty and staff, based on demographic and socioeconomic prevalence estimates [41,42,43,44,45,46,47,48]. Low and high values reflect uncertainty bounds on alternate prevalence assumptions. Percentages represent the proportion of the total population; absolute numbers represent the corresponding estimated number of individuals. ASCVD: atherosclerotic cardiovascular disease; CHD: coronary heart disease; MI: myocardial infarction.

Incremental costs to improve nutrition for such a community approach can be low. Refined flour, for instance, is inexpensive. Even the cost of packaging for a loaf of bread (7% of cost) is greater than the farm value of commodity flour (5% of cost) [49,50]—a 75% premium paid for flour cost would add only ½ cent per serving. (The premium paid for increased-fiber wheat with this approach exists to ensure that wheat from increased-fiber cultivars are delivered to the target community. This cost is not required when the goal is for increased fiber to benefit public health, in general.) Wheat provides an even smaller share of the cost of many community favorites, such as macaroni and cheese.

Other low-cost interventions can be incorporated to multiply the benefits of nutrition reformulation. Depending on the production set-up, adding a cooling step to increase resistant starch may cost nothing. Some fiber-dense ingredients used as additives such as legumes, pea fiber, and flaxseed can also be cost neutral to add 5 g fiber to community favorites such as macaroni and cheese, as the fiber-rich options substitute for more expensive ingredients.

Increased-fiber options that encourage consumers to change their food choices, such as medically tailored meals (MTMs), tend to be considerably more expensive. To influence consumers with coupons, for instance, a large enough discount must be provided to impact behavior: fruit and vegetable coupon programs, for instance, allocate USD 1 a day per person [51], and the Michigan producer prescription also provides USD 1 coupons [52]. When enhancing nutrients in current foods, the trade-off is that the nutrient increase per individual is likely to be smaller, to minimize the impact on foods’ organoleptic properties.

#### 3.2.2. Financing Community Food Supply Intervention

While costs to increase nutrient density of foodservice options can be neutral or minimal, someone must pay to implement change. To maintain the current price that consumers pay for staple foods, another entity must provide the financial incentive. The cost may be low enough that service providers or donors would choose to cover it for public relations, or to market their now-healthier approach. Evidence from school-based nutrition interventions demonstrates that government-driven food demand can also facilitate large-scale adoption of healthier formulations without reducing participation or increasing waste, while maintaining foodservice operators’ economic viability [53,54].

The systematic coverage of cost by healthcare insurance provides an opportunity to potentially pilot and then scale nutrition-focused, science-driven protocols that materially impact the cost structure and profitability of healthcare funders. The benefits to health insurers would be greatest for those with more stable relationships—either with specific individuals or communities—such as private institutions [55,56,57], governments [58,59], or community insurers [60]. From an insurers’ profitability perspective, immediate cost reductions associated with improved outcomes for individuals afflicted by chronic conditions would be more compelling than disease prevention, which only impacts costs longer term.

Pilot programs can be implemented to identify the most compelling approach in a particular geography. Options could include policy waivers (e.g., under Medicaid Policy Section 1915 [61]); funding from programs that explicitly support preventive care [62,63]; or community contracts with insurers that reward improved health outcomes [64,65]. In each case, the primary focus on enhancing community health at low incremental cost can establish a systematic foundation on which to expand effective interventions.

In this community-focused supply intervention, insurers cover the financial cost of incentives in order to benefit from the better health outcomes that result when individuals with chronic conditions increase their fiber intake.

Together, these community-focused and the commodity-focused approaches demonstrate how agricultural innovation and food processing can be integrated with economic incentives to create scalable, system-level mechanisms for improving diet quality. The potential population-level health and economic implications of these pathways are evaluated in the following section.

## 4. Impact

With the approach assessed in this article, low-cost ingredients like wheat flour can reduce high-cost chronic disease and improve health outcomes, with no clinically meaningful adverse effects expected. For example, the U.S. wheat crop, feeding hundreds of millions of people across the nation and globally, is valued at USD 10–20 billion a year (USD 13 billion in 2023), 60 times smaller than the annual USD 800 billion in U.S. healthcare costs for the cardiovascular disease, type 2 diabetes, and colorectal cancers [66,67] that wheat’s fiber can help reduce.

Although the impact of individual food choices depends on overall dietary patterns, fiber remains an under-consumed nutrient of concern, and the U.S. Department of Agriculture recognizes that increasing fiber intake—an outcome which this paradigm aims to achieve—advances public health. Research overwhelmingly shows that increased dietary fiber intake has a positive health impact [68,69,70,71,72,73]. Because fiber intake varies across a population in a continuous distribution—some take a small amount, others moderate, and a few have high intake—any increase in intake for each individual in the population ensures that at least some individuals benefit. As long as there are at least some intake levels at which additional fiber improves health, then raising intake for the bulk of all individuals in the population will improve health for at least some individuals, and thus, in aggregate, improve overall public health. These comparative statics are commonly applied and analytically supported in many scientific areas [57,74,75]. (Monotonicity follows from monotone comparative statics under increasing differences (Milgrom & Shannon, 1994 [74]). Continuity of the outcome with respect to parameters follows from Berge’s Maximum Theorem. Aggregating over a continuous distribution of heterogeneity yields an aggregate response equal to the population cumulative distribution function, hence continuous and increasing (see [75] on smooth choice probabilities under continuous heterogeneity).)

**Figure A1:**
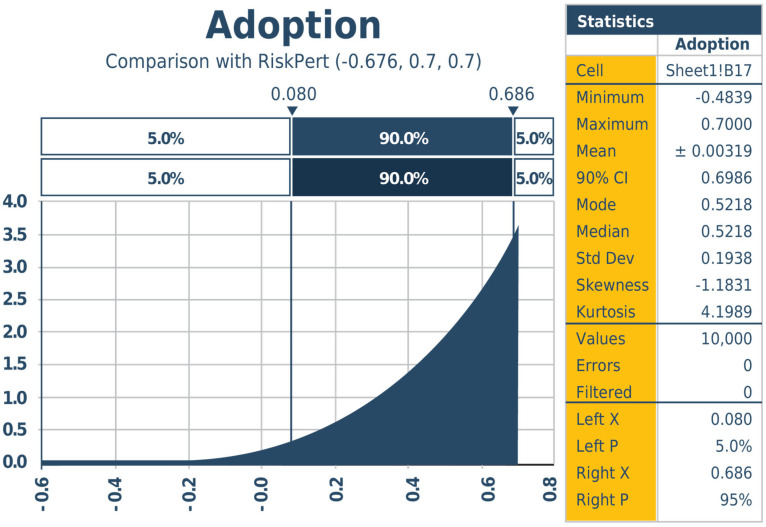
Adoption Probabilities and Uncertainty.

Meta-analysis was employed to model the benefits of the initial increased-fiber wheat on the U.S. population, using a comparative risk assessment framework. Meta and @Risk software analysis frameworks and results are summarized in Appendix A Table A1 and Figure A1, based on U.S. data for demographics and baseline disease incidence. The adoption probabilities in Figure A1 drive production, health, and economic outcomes. Peak projected U.S. implementation benefits include USD 12 billion in annual healthcare cost savings, 6 million more Americans avoiding medical conditions, and over 60,000 deaths prevented annually, excluding any benefits from increased fiber in exported wheat. (Provided the midpoint of the referenced risk reduction rates, USD 12 billion is the lower bound for peak annual cost reduction. If there is no cost reduction associated with improved outcomes for individuals with the chronic conditions, USD 12 billion results from the shrunk disease population after a generation. If improved outcomes correspond with equivalent percent reduction in treatment costs, for example, that amount is the “immediate” cost reduction, increasing to more than an additional 90% of the initial cost reduction over the coming generation.).

## 5. Coordination and the Path to Success

**Figure 6:**
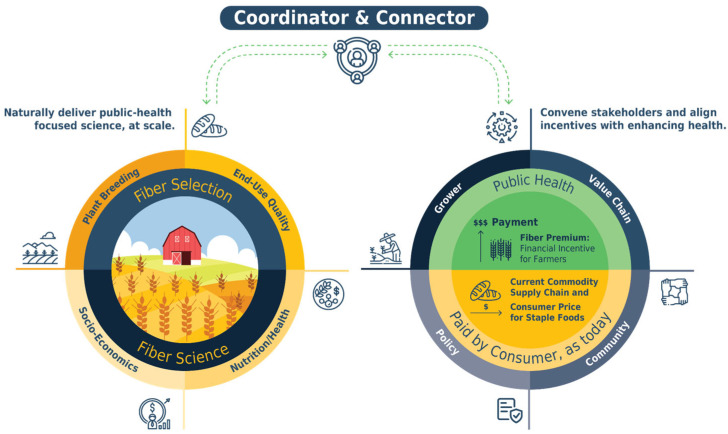
The left circle shows how plant breeding and fiber science increase dietary fiber while assessing farming, milling, bread-making, health, and economic impacts. The arrows show continuous feedback and improvement loop. The right circle shows public health stakeholders supporting incentives, such as grower premiums. The “little hinge” coordinates.

The various low-risk food technologies and market-based strategies and policies that can deliver increased-nutrient opportunities share one characteristic: they each require aligned activity. In Figure 6, the coordinating body that supports alignment is represented as the “hinge”.

The left side of Figure 6 highlights the importance of aligned, credibly neutral, scientifically rigorous, and policy-connected scientific efforts. A governance model akin to the United Nations Intergovernmental Panel on Climate Change (IPCC) could accelerate the development and adoption of nutrient-enhanced food interventions. With an annual budget of about USD 6 million [76], the IPCC, supported by its administration and protocols, acts as a “hinge” that swings the “big door” for the thousands of scientists with varied expertise it brings together to collaborate. Participants produce periodic assessments and special reports about insights from climate science, with engineers exploring emissions; climatologists examining atmospheric change; economists quantifying consequences in multiple metrics; synthesis teams reporting on five integrated “Reasons for Concern”; and additional communication for technical and non-technical audiences. While scientists are not paid for their time (aside from travel expenses being reimbursed), they participate in the IPCC’s efforts because the IPCC’s integration provides context for their individual work. It clarifies its broader implications and provides roadmaps for the gaps in knowledge where the value of new information and understanding will be maximized. Similarly, the framework proposed here aligns plant breeding, end-use quality, nutrition/health, and socioeconomic efforts for nutrition enhancement, ensuring increased nutrition alongside continued improvement in crop performance and food production, with limited supply chain disruption. Up until now, health outcomes have not been linked strongly enough to agricultural production priorities with scalable and aligned science, despite the opportunity.

Effective governance could similarly support the economic innovation described in this article, as seen in the governed activities in the right circle of Figure 3. The key is bridging the gap between public health and agriculture, allowing those who receive value from improved public health to financially support the supply of foods with improved nutritional quality. This could be achieved by convening global, national, and community stakeholders to identify concrete opportunities for collaboration. These include policy makers responsible for health, agriculture, and food systems; public and private for-profit interests, including farmer and food supply chain groups, food organizations, and, in some contexts, health insurance companies; and rural and urban communities, whose needs, preferences, and constraints shape adoption and impacts and with whom market resistance and unintended shifts in diet can be evaluated. All of these stakeholders would either benefit from public health or from receiving financial incentives to deliver the increasingly nutrient-dense foods that support that health. By coming together, they can find their common interest.

## 6. Hurdles and Limitations

For any new proposed public health program, a number of hurdles and potential surprises must be considered. Multiple participants in the food supply chain and in public health must shift their focus for such a program to maximize impact. Challenges in implementation and unintended consequences are possible. In particular, the paradigm described above cannot rule out unintended shifts in consumers’ diets, despite not primarily relying on such changes. Policy makers would have to stay aware and reactive. Moreover, nutritional programs must be validated for success—a step that is needed. In deciding the extent to which to adopt this approach, policy strategists must consider the likelihood of a breakthrough in benefits, the potential for unintended impacts, and the realistic promise of ongoing, incremental improvements through current nutrition interventions.

## 7. Conclusions

By enhancing nutrients in widely consumed staple foods, the HEAL approach reframes nutrition improvement as a systems-level intervention rather than one dependent on individual behavioral change.

The HEAL approach leverages the low-cost, global reach, and low-margin structure of food systems, allowing modest incentives to efficiently shift production and processing toward healthier foods. Parties that value public health—notably health insurers, communities, nonprofits, and governments—can choose to help finance these nutrition improvements using a portion of the healthcare savings generated through reduced diet-related disease burden. Targeted mechanisms further support the rigorous evaluation of evidence and communication with stakeholders, enabling the ongoing alignment of science, markets, and policy.

This approach aims to mutually benefit stakeholders across multiple time horizons. With food production and processing formally linked with HEAL, both sectors would be strengthened. Agricultural competitiveness is improved by innovating toward improving the healthfulness of existing products [77], with healthcare costs reduced. With savings from healthcare leveraged to cover the limited incremental cost, national populations would benefit across all socioeconomic groups, with no change in consumer price for staple foods. Health outcomes for chronic disease would soon begin to improve, with projected millions fewer cases of chronic disease and billions in reduced annual healthcare costs in the medium to long term.

Increased-fiber wheat serves as the primary case study to illustrate this approach, which is applicable across nutrients, technologies, and staple foods. Although individual nutritional changes may be modest, in order to increase nutrition at the population scale without compromising taste or cultural relevance, their simultaneous delivery to millions of consumers—and the opportunity to combine incremental improvements—would amplify their impact. The technical capacity to implement such changes already exists; the remaining challenge is in coordinating the stakeholders.

## Figures and Tables

**Table 1:** Estimated prevalence of selected cardiometabolic conditions: Delaware State University 7000 students, faculty, and staff.

	Low %	High %	Low Prevalence	High Prevalence
Prediabetes	21%	27%	1460	1900
Diabetes Type 2	4%	8%	300	550
Heart Disease History (CHD/Angina/MI, etc.)	1%	2%	54	150
High 10-Yr ASCVD Risk (≥20%)	1%	2%	75	119
Other Forms of Cardiovascular Disease	25%	32%	1715	2271
Total Conditions	51%	71%	3604	4990

## Data Availability

No new data were created or analyzed in this study. Data sharing is not applicable to this article.

## Appendix A

**Table A1:** The following outlines the financial valuation assumptions and methodology leading to the factors reported in the body of this paper, using commonly applied net present value of future values methodology.

Parameter	Specification	Source/Validation
BASELINE CONDITIONS		
Target population	U.S. adults (≥18 years; n = 258 M)	U.S. Census Bureau, 2023 [78]
Baseline fiber intake	15 g/day (mean); 5% meet recommendations (≥28 g/day)	Zhuo et al. 2023 [2]; NHANES 2017–2020 [79]
Wheat consumption	127 g/day per capita	USDA ERS, 2023 [80]
INTERVENTION SCENARIO		
Fiber increase target	+2.5 g/day via high-fiber wheat	Deehan et al. 2024 [68] (physiological feasibility)
Adoption rate	70% replacement of conventional wheat products over 10 years	USDA ARMS adoption curves for biofortified crops
Fiber content enhancement	+30% fiber in refined flour	Shewry et al. 2024 [19]
HEALTH IMPACT MODEL		
Modeling framework	Comparative risk assessment (CRA)	Global Burden of Disease Study methodology
Risk reduction per 2.5 g fiber	- CVD: 1–3% - T2D: 3–4.5% - CRC: 1.5–3.5%	Canene-Adams et al. 2022 [22]; The InterAct Consortium [23]; Huxley et al. 2013 [24].
Outcome metrics	Cases prevented, deaths avoided, productivity gained	CDC Wonder Database; IHME DALY weights
**ECONOMIC MODEL**		
Modeling framework	Cost-of-Illness (COI) + Societal Perspective CEA	WHO Guide to Cost-Effectiveness Analysis
Cost parameters	- CVD: USD 342 billion/year - T2D: USD 332 billion/year - CRC: USD 24 billion/year (used)	CDC Chronic Disease Data; AHRQ MEPS
Savings calculation	Total cost reduction = total number of cases prevented * average lifetime treatment cost per case	Miller et al. 2022 [4]
Discount rate	9.71% annual. Rather than 3%, as per the U.S. Panel on Cost-Effectiveness in Health, the higher rate is used to be competitive with private investment	U.S. Panel on Cost-Effectiveness in Health
**SENSITIVITY RANGES**		
Adoption rate	RiskPert (-0.676,0.7,0.7) 5% = 7.96%, 50% = 52.19%, 95% = 68.59%	Probabilistic sensitivity analysis
Risk reduction	±25% of central estimate	Monte Carlo simulation (10,000 iterations)
